# Impact of Watermelon (*Citrallus lanatus*) on Male Fertility

**DOI:** 10.5935/1518-0557.20220075

**Published:** 2023

**Authors:** Damilare E. Rotimi, Rotdelmwa M. Asaleye

**Affiliations:** 1 SDG 03 Group - Good Health & Well-being, Landmark University, Omu-Aran 251101, Kwara State, Nigeria; 2 Department of Biochemistry, Medicinal Biochemistry, Nanomedicine & Toxicology Laboratory, Landmark University, PMB 1001, Omu-Aran-251101, Nigeria

**Keywords:** male infertility, sperm, testis, testosterone, watermelon

## Abstract

Plants have been used in various regions of the world to treat various medical conditions including male infertility. The review aims to evaluate the pharmacological effects of watermelon consumption in improving male fertility and sexual function. Watermelon is a popular fruit consumed around the world for its diverse nutritional and health-promoting qualities. This study showed the mechanism via which watermelon enhances male fertility as it was reported for improving semen quality, reversing erectile dysfunction, enhancing testicular redox status, as well as improving gonadotropin secretion. These activities have been linked to their constituents as it contains vitamins and phytochemicals such as phenols and certain flavonoids that contribute to their antioxidant properties. Watermelon has also been noted to possess antimicrobial, anti-helminthic, antioxidant, antidiabetic, anti-inflammatory, and antihypertensive properties that may contribute to its therapeutic use.

## INTRODUCTION

Infertility is characterized by the inability of a couple to become pregnant after a year of frequent intercourse without the use of contraceptives (Akhondi *et al*., 2019). Infertility can lead to stress, emotional pain, disappointment, frustration, fear, and anxiety for affected couples. Globally, over 50 million couples report being affected by fertility challenges, with about 30-50 % citing male reproductive disorders as the underlying issue (Fainberg & Kashanian, 2019; Olaolu *et al*., 2018). Major causes of male infertility include reduced semen quality, dysfunctional hypothalamic-pituitary-gonadal axis, infections, and endocrine disorders (Gunes *et al*., 2015; Iyiola *et al*., 2019; Kayode *et al*., 2020a; Musavi *et al*., 2018; Nwonuma *et al*., 2021; Olaolu & Rotimi, 2017; Pourmasumi *et al*., 2017). Potential treatment options include surgical interventions, hormone therapy, and specialized assisted reproductive technology (ART). Some examples of ART include intrauterine insemination (IUI), in vitro fertilization (IVF), intracytoplasmic sperm injection (ICSI), gamete intrafallopian transfer (GIFT), zygote intrafallopian transfer (ZIFT), and third-party fertilization (donated eggs, sperm, uterus, and embryos) have all been used to treat male fertility issues in modern medicine (Musavi *et al*., 2018). Like all medical procedures, ART is associated with limitations of the application. Some examples of limitations of ART include mental and physical stress, surgical complications, and significant cost. Beyond this, patients may not qualify for specific therapies based on underlying medical conditions. These limitations lead patients to search for alternative interventions which are safe, affordable, and effective therapies. One category of alternative interventions that might be considered by patients is herbal remedies. Plants (herbs) have been used in various regions of the world to treat male infertility and a range of other medical conditions (Nwonuma *et al*., 2021). Many male fertility issues have been reported to be treated using herbal remedies, including improving sperm quality, sexual dysfunction, and erectile/ejaculatory problems. Celery, fennel, black seed, German chamomile, saffron, Vitex, fumitory, Oregano, and carrot are some of the plants that may promote male fertility (Dutta & Sengupta, 2018; Kayode *et al*., 2020b; Malviya *et al*., 2016; Olaolu *et al*., 2021; Vyas *et al*., 2018).

### Ethnobotanical use of watermelon

Watermelon (*Citrallus lanatus*) is another potentially useful plant used for a variety of medical conditions. Concerning male fertility issues, it has also been reported to improve semen quality, erection, redox status, sexual function, and increase testosterone and gonadotropin secretion. Together, these features of watermelon may improve the fertility rate in male consumers (Khojasteh *et al*., 2016).

Watermelon is consumed for many reasons. Some of the features that make watermelon a popular fruit include its appealing color, distinctive flavor, and high water content, which can help to relieve thirst (Erhirhie & Ekene, 2013; Maoto *et al*., 2019; Renner *et al*., 2017). The fruit has a thick smooth outer rind [exocarp] and a pleasant, nutritious, juicy, fleshy interior [mesocarp and endocarp]. Sucrose and glucose make up about 20–40% of the total sugars in a mature watermelon, whereas fructose makes up 30–50% (Erhirhie & Ekene, 2013). The rind is normally tossed away; however, it is edible and can be eaten as a vegetable, fed to animals, or used as fertilizer (Ibrahim *et al*., 2017). In addition, watermelon rinds can be fermented or blended to be served as juice. Therapeutically, rinds have been administered in conditions involving alcohol intoxication and diabetes. When completely ripe or almost rotting, the fruits are consumed as a febrifuge. The fruit has high potassium content, making it more suitable for treating potassium deficiency and kidney stones (Deshmukh *et al*., 2015). Watermelon roots can be strongly purgative and therefore used as an emetic at high doses (Erhirhie & Ekene, 2013). The seeds may also have an antihypertensive property and be useful for reducing blood pressure. In addition, watermelon seeds have anti-helminthic properties and are occasionally prescribed for the treatment of helminthic infections. Experimentally, tapeworms and roundworms were observed to be inhibited by a fatty oil contained watermelon seeds, as well as aqueous or alcohol extracts. The seeds have also been used to extract tar, which is used to cure scabies and to tan the skin (Nasiru *et al*., 2019).

Watermelon consumption has rapidly gained popularity in developing countries and is highly recommended locally due to its numerous reported health benefits. The popularity of watermelon is related to, at least in part, the fact that it is fat-free, cholesterol-free, sodium-free, and high in minerals and phytochemicals (Mariya *et al*., 2020).

The chemical composition present in watermelon has been shown to decrease low-density lipoprotein (LDL) and high-density lipoprotein (HDL) in the cell membrane (Maoto *et al*., 2019). It has been suggested that watermelon could be used for weight loss (Hassan *et al*., 2011). In Northern Sudan, watermelon is used to treat burns, swelling, rheumatism, and gout, and as a laxative (Hassan *et al*., 2011). In Senegal, watermelon is used as a purgative, while in Nigeria, it is used to cure diarrhea and gonorrhea. Numerous epidemiological studies have reported its effectiveness in the treatment of cardiovascular disease (CVD) (Naz *et al*., 2014). As a result, watermelon consumption has been linked to a variety of therapeutic benefits, including a reduced risk of age-related neurodegenerative disorders and certain cancer types. Furthermore, watermelon is a good source of citrulline, a nonessential amino acid that is also a nitric oxide booster (Naz *et al.*, 2014; Njoya *et al*., 2019; Oyelakin & Atilola, 2015).

### Phytoconstituents of watermelon

Watermelon is a robust source of vitamins, minerals, and other important substances that may contribute to its pharmacologic applications. Vitamins provided through watermelon consumption include thiamine, riboflavin, niacin, and folate. Beyond this, vital electrolytes including potassium, magnesium, calcium, phosphorus, and iron are also found in watermelon. Together, these nutrients are known to function as cofactors for numerous cellular enzymes, play important roles in cell signaling, contribute to the maintenance of cellular architecture, and promote healthy cell differentiation (Aderiye *et al*., 2020). In addition, watermelon has a greater antioxidant content than other fruits including tomatoes, strawberries, and guavas (Maoto *et al*., 2019; Poduri *et al*., 2013). Carotenoids found in watermelons such as lycopene and β-carotene are responsible for the watermelon’s red and orange hues, respectively. Cucurbitacin, triterpenes, sterols, and alkaloids are bioactive chemicals found in watermelon (Zia *et al*., 2021). Taken together, these bioactive compounds have been linked as potentially therapeutic in issues related to male fertility. Given these inherent properties of watermelon, this review aimed to evaluate the effects of watermelon on male fertility, taking into account the risks and limitations of chemical medications and surgical methods.

### The effect of watermelon on male fertility

The male reproductive system is particularly prone to structural and functional changes that result in male infertility. Therefore, there is a need to look for therapeutic options that can improve or mitigate the factors responsible for the change in the male organs.

#### Antioxidant effects on improving fertility

**Figure 1:**
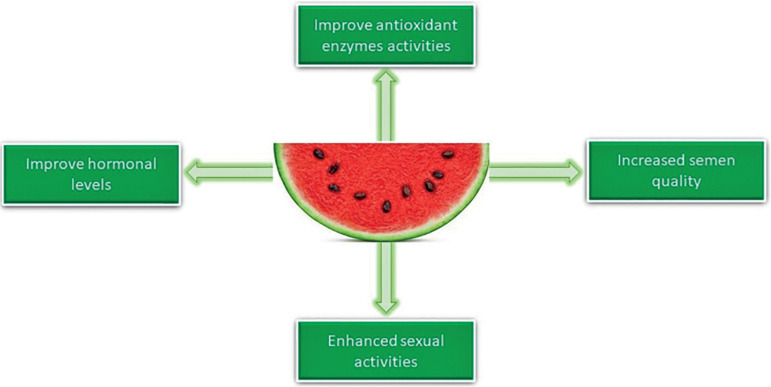
The therapeutic effect of watermelon on male fertility.

Watermelon contains lycopene, which is a powerful antioxidant. It also contains several phytochemicals including phenols and flavonoids, which could be responsible for its therapeutic role in reproductive pathophysiology (Figure 1; Table 1). One of the greatest known attributes of most flavonoids is their antioxidant properties (Akintunde & Thomas, 2021). Flavones and catechins are among the most potent flavonoids that protect the body against reactive oxygen species damage (Panche *et al*., 2016). Carotenoids found in watermelon including β-carotene, lutein, and lycopene provide the consumer a healthy boost in antioxidant levels. Experimentally, watermelon consumption has been associated with increased plasma antioxidant levels. One epidemiologic study in China associated the use of watermelon in the diet with a lower incidence of cancers (Panche *et al*., 2016).

**Table 1:** The effect of watermelon on male reproductive function.

Reference	Animal used	Plant part used and form of extract	Route of administration	Dosage	Duration of Treatment	Effect of Watermelon
Onyeso *et al*. (2016)	Wistar rats	Seeds; methanolic extract	NM	100 and 200 mg/kg	30 days	*Increase in serum level of testosterone *Increase the sperm motility, count, morphology, viability *Improved histological structure
Amedu & Idoko (2016)	Wistar rats	The inner part of the fruit	Intraperitoneally	Juice; NM	8 weeks	*Increased level of testosterone, FSH, and LH *Increased level of sperm count, motility, concentration, and morphology
Kolawole *et al*. (2017)	Wistar rats	Rind; Methanolic Extract	Orally	200 mg/kg	35 days	*Enhanced sperm count *Increased all reproductive hormone levels *non-significant increase in sperm motility, percentage of spermatocytes with normal morphology, and percentage of live spermatocytes, but decreased percentage of dead spermatocytes.
Khaki *et al*. (2014)	Wistar rats	Seed; hydro alcoholic extract	Oral gavage	30 mg/kg	28 days	*Serum testosterone was enhanced *Enhanced sperm quality
Daramola *et al*. (2018)	Wistar rats	Fruit; aqueous extract	Orally	100 and 200 mg/kg	30 days	*Increased the Follicle-stimulating hormone *Decreased the malondialdehyde level and increased the superoxide dismutase level. * Improved testicular histology
Akhuetie *et al*. (2018)	Wistar rats	Seed; crude powder and ethanolic extract	Orally	200mg/kg	28 days	*Reduced sperm morphological abnormalities *Testicular histology showed numerous closely packed seminiferous tubules with normal architecture containing spermatogenic cells
Kolawole *et al*. (2019)	Wistar rats	Rind; Hydromethanolic Extract	Orally	500 mg/kg	42 days	*Increased sperm count and reproductive hormone concentrations *increase in superoxide dismutase concentration and decreased malondialdehyde level
Pratama *et al*. (2021)	Wistar rats	Rind; ethanolic extract	Orally	100, 200 and 400 mg/kg	52 days	At 200 and 400 mg/kg, there was an increase in the number of Sertoli and spermatogenic cells.
Odo *et al*. (2021)	albino rats	Seed; ethanolic extract	Orally	200 mg/kg	8 weeks	*Increased testicular weight *Increased Testosterone level, *Increased sperm motility, *Increased gonadal sperm, and extragonadal sperm reserves *Optimum histoarchitectural protection of the seminiferous tubules
Awaad & El Gamel (2020)	Sprague Dawley Strain	pulp and seed; methanol extract	Orally	100 and 200 mg/kg	28 days	*Increased Testosterone, follicles stimulating hormone and luteinizing hormone in all groups tested *Decrease in serum prolactin hormone *Improved testicular histoarchitecture
Onyinye & Emeka (2019)	adult Wistar rats	Seed; ethanolic extract	NM	500 and 1000 mg/ kg	14 days	*Enhanced libido *Increased mounting and intromission frequencies *Enhanced intromission and ejaculation latencies, *Decreased mounting latency and post-ejaculatory interval. *Serum testosterone and luteinizing hormones were increased. *improved histoarchitecture of the testes and hypothalamic sections.

Khaki *et al*. (2013) suggested that the antioxidant properties of watermelon seeds could protect sperm DNA from free radicals, enhance blood-testis barrier integrity, and protect other essential components of the reproductive system from oxidation, ultimately improving sperm quality and, as a result, boost male fertility (Khaki *et al*., 2013; Oseni & Okoye, 2013).

Choudhary *et al*. (2015) also reported that watermelon contains a high vitamin C content. Some antioxidant vitamins, particularly tocopherol and ascorbic acid, have been found in the rind of watermelon (Johnson & Walcott, 2013). The presence of Vitamin C has been shown to preserve human sperm by neutralizing hydroxyl, superoxide, and hydrogen peroxide radicals, as well as inhibiting sperm agglutination. As a result, the presence of vitamin C is believed to aid in peroxidation, resulting in better sperm morphology and viability (Johnson & Walcott, 2013).

Many studies have reported the various antioxidant effects of watermelon on male reproductive processes. According to Kolawole *et al*. (2019), co-administration of nicotine and a hydromethanolic extract from watermelon rind resulted in improvements in semen quality as well as increased testosterone and gonadotropin secretion in male Wistar rats. The administration of watermelon was able to ameliorate the effect produced by nicotine on the male reproductive organ in male Wistar rats. This is consistent with the findings of Kolawole *et al*. (2017) and Daramola *et al*. (2018) who found that the watermelon extract reduced lead acetate and arsenic toxicity, respectively. Beyond these, Onyinye & Emeka (2019) reported that 200 mg/ kg of watermelon seed was able to reduce the toxicity induced by alloxan. The effect of reducing toxicity may lead to improved sperm motility, count, morphology, viability, and testosterone.

Khaki *et al*. (2013; 2014) found that daily injection of the hydro-alcoholic extract of watermelon seeds (30 mg/ kg daily for four weeks) enhanced sperm motility viability, and total count (Khaki *et al*., 2013; 2014). Phenols found in watermelon have also been proposed to be important in detoxifying the body. The phenol content of watermelon has been suggested to chelate arsenic and enhance its excretion from the body (Oseni & Okoye, 2013). Phenols might also activate endogenous antioxidant enzymes present in the epididymis (Oseni & Okoye, 2013). In this study, both 100 and 200 mg/kg of extract ameliorated the effects of arsenic on sperm viability and morphology (Daramola *et al*., 2018).

#### Other mechanisms of improved fertility

According to Onyinye & Emeka (2019), administration of watermelon ethanolic seed extract significantly improves sexual behavior as evidenced by increased mounting and intromission frequencies, ejaculation latency, and intromission latencies, as well as a shorter post-ejaculation interval. In addition, watermelon ethanolic seed extract supplementation was linked to an increase in serum sex hormones in male rats (Onyinye & Emeka, 2019). Following watermelon ethanolic seed extract treatment, testicular tubular size, compact seminiferous tubules, and the number of spermatozoa in the lumen of the seminiferous tubules increased significantly. These findings are consistent with those of Munglue *et al.* (2014), who found that the presence of pharmacologically active compounds in watermelon increased semen quality and sexual activity in male rats.

Watermelon seeds contain significant quantities of precursor substrates like arginine and citrulline, both of which are involved in the production of nitric oxide, a powerful vasodilator (Arise *et al*., 2016). Nitric oxide (NO) is an endogenous chemical that regulates the turgidity of penile erection by upregulating guanosine monophosphate (Aguayo *et al.*, 2021). Therefore, the consumption of watermelon provides the user with sufficient nutrients to synthesize NO, given that all other mechanisms of production are intact. Beyond this, Munglue *et al*. (2014) discovered that watermelon extract had aphrodisiac properties in rat models. This might be related to the presence of citrulline, a phytonutrient in the seed (Munglue *et al*., 2014). Citrulline has the additional benefit of being converted to arginine, which has been shown to increase sperm count in males (Munglue *et al*., 2014).

#### Safety

The general belief is that natural or plant medicines may be safe mainly because of anecdotal usage without significant amounts of reported toxicities associated with their use. Despite this, generalizations can have potential safety implications for patients (Ezuruike & Prieto, 2014). Studies have indicated that many herbs used in traditional medicine may produce adverse effects. Data from Oyenihi *et al*. (2022) revealed that consumption of watermelon seed may lead to tissue-related toxicity specifically in the testes. This finding raises a safety concern for humans, especially chronic consumers. The consumption of these seeds at high doses could be detrimental to male reproductive function as evidenced by a significant decrease in sperm morphology in rats (Oyenihi *et al.*, 2022). Additional dose-related studies and an extended experimental period are required to identify the safe dosing range for watermelon seed to maximize its potential health benefits.

Conflicting results have been reported for the impact of watermelon seed consumption on testes and sperm. A study by Ebeye *et al*. (2015) reported that the aqueous extracts at 100 and 300 mg/kg doses of watermelon seed led to dose-dependent testicular and Leydig cell hyperplasia, interstitial hydrolysis, and decreased spermatogenesis. In another earlier study, the hydro-alcoholic extract was administered at a 55 mg/kg dose and reported an improvement in sperm motility and viability (Khaki *et al*., 2013). Variations in the concentrations of tested chemicals could be responsible for these findings. Other constituent chemicals found within watermelon may also confound experimental outcomes. For example, saponins and certain alkaloids have been shown to interfere with spermatogenesis. The observed effects of the watermelon seed diet study may be mediated in part by these alkaloids and saponins amongst others. These compounds can elicit direct cell toxicity and reduce sperm quality via disturbances in Leydig cell function, vacuolation of the spermatozoa plasma membrane, and modification of ionic transport across the membrane (Singh *et al*., 2014). Cyanide and oxalate are some other potentially toxic antinutrients identified in watermelon seeds although at lower concentrations than other antinutrients. A study by Johnson & Walcott (2013) reported a significantly higher hydrocyanide concentration (HCN) (3-fold increase) in fresh watermelon seeds (1.47±0.03 mg/100 g of watermelon seed) than in the pulp and rind.

### Future Research

In many parts of Africa (especially sub-Saharan Africa), Citron watermelon serves to combat food and nutrient insecurity. However, the consumption and utilization of this food source remain low, which could be attributed to the dearth of knowledge of its medicinal, nutritional, and pharmacological properties. There is a need to explore and further document the ethnopharmacological uses of watermelon as its utility is scarcely reported and under-exploited. Several phytochemicals have been identified in watermelon, although limited research has been focused on the identification, isolation, and quantification of specific phytochemicals. Under-researched chemicals with potential therapeutic value include cucurbitacins and related glycosides. Thus, there is a need to identify the various phytochemicals in watermelon to quantify and isolate their biological activities and mechanism of action for medicinal applications. Further studies are required *in vivo* and *in vitro* to form conclusions about the efficacy of the use of these chemicals, especially as it relates to male infertility. Increased evidence of utility would enable the reliable utilization of watermelon in the treatment of male infertility. Moreover, due to the limited number of clinical studies, there is no conclusive role for watermelon in fertility-related medical management. Clinical studies with large sample sizes, varied duration of administration, comparison with safe drugs, and the determination of the exact molecular mechanism are recommended.

## CONCLUSION

Watermelon has played an important dietary and medicinal role throughout the history of mankind. However, the mechanism for its therapeutic actions on male reproduction remains unknown. The antioxidant properties of watermelon have been shown to improve sperm quality, male sexual dysfunction, and to improve testicle function.
